# Effectiveness of combined salmon calcitonin and aspirin therapy for osteoporosis in ovariectomized rats

**DOI:** 10.3892/mmr.2015.3637

**Published:** 2015-04-16

**Authors:** JINSONG WEI, JIAN WANG, YAN GONG, RONG ZENG

**Affiliations:** Department of Orthopedics, Affiliated Hospital of Guangdong Medical College, Zhanjiang, Guangdong 524001, P.R. China

**Keywords:** osteoprotegerin/receptor activator of nuclear factor κB ligand (RANKL)/RANK system, osteoporosis, salmon calcitonin, aspirin

## Abstract

The objective of the present study was to assess the effectiveness of combined salmon calcitonin (sCT) and aspirin [acetylsalicylic acid (ASA)] treatment in an ovariectomized (OVX) rat model of postmenopausal osteoporosis. Following 12 weeks of treatment, therapeutic efficacy was assessed by evaluating changes in the biochemical and biophysical properties of bone (n=8 rats per group). Serological markers of bone metabolism were measured by ELISA; bone mineral densities (BMD) by dual energy X-ray absorptiometry; bone biomechanics of the femur and lumbar vertebrae by three-point stress test; trabecular bone morphology of lumbar vertebrae by hematoxylin and eosin staining; messenger RNA expression levels of osteoprotegerin (OPG) and receptor activator of nuclear factor κB ligand (RANKL) in bone marrow cells by reverse transcription-quantitative polymerase chain reaction and OPG and RANKL protein expression levels in the proximal tibia were analyzed by immunohistochemistry. Compared with treatment by sCT or ASA alone, combined treatment (sCT+ASA) increased BMD, improved femur bone strength, normalized trabecular network architecture and morphology, and increased mRNA and protein expression of OPG, while reducing the expression of RANKL. Collectively, these results demonstrated that combined treatment (sCT+ASA) of osteoporotic symptoms in OVX rats was more effective than treatment with sCT or ASA alone. Furthermore, these two drugs appeared to alter the expression of two distinct factors in the OPG/RANKL/RANK system, suggesting that their effects may be synergistic. Since sCT and ASA are currently approved for use in humans, the results of the present study suggest that the safety and efficacy of sCT+ASA combined therapy for postmenopausal osteoporosis should be assessed in clinical trials.

## Introduction

Osteoporosis is an age-associated systemic disease that is characterized by a progressive loss of bone mass, decreased bone strength and increased fracture risk (1). Onset of the disease often has no warning sign or symptoms, and therefore may remain undiagnosed until the pain induced by an osteoporotic fracture attracts attention. Normal maintenance of bone tissue strength and integrity is a dynamic process that requires constant remodeling of the mineralized bone matrix by two opposing processes. New bone tissue is formed by specialized osteoblast cells (bone formation), and existing bone matrix is broken down by osteoclast cells to be re-absorbed by the body (bone resorption). If these processes are in balance, the bone mass is maintained. However, if osteoblast activity is increased compared with osteoclast activity, then a net gain in bone mass will occur. In osteoporosis, the loss of bone mass, commonly detected as a decrease in bone mineral density (BMD), is the result of an imbalance between the rates of bone formation and resorption.

Postmenopausal women are particularly susceptible to this disorder of bone metabolism. During child-bearing years, estrogen aids the maintenance of bone mass in women by stimulating osteoblast activity. The reduced estrogen levels in postmenopausal women lead to reduced rates of bone formation by osteoblasts, resulting in a net increase in bone resorption (1). In addition, decreased proliferation of osteoblasts in postmenopausal women results in enhanced differentiation and hyperactivity of osteoclasts (OCs), resulting in bone loss. Considerable evidence indicates that the osteoprotegerin (OPG)/receptor activator of nuclear factor κB ligand (RANKL)/RANK system is necessary for osteoclast development (2,3). RANKL, also known as tumor necrosis factor superfamily member 11 (TNFSF11), is secreted by T lymphocytes, B lymphocytes, stromal cells and osteoblasts (4,5). RANKL binds to a membrane-bound receptor (RANK or TNFRSF11A) on the surface of osteoclast precursor cells, promoting the differentiation, maturation and activity of OCs. The soluble receptor OPG (TNFRSF11B), secreted by stromal and osteogenic cells (including osteoblasts), competes with RANKL to bind to RANK, thereby preventing the differentiation and activation of OCs (3,6).

Numerous drugs have been investigated for use in the treatment of osteoporosis for postmenopausal women with elevated bone resorption rates. Hormone replacement therapy may inhibit bone loss; however, long-term use of hormone treatment may cause venous thromboembolism, coronary heart disease and stroke (7–9). Bisphosphonates (BPs) have been reported to have high affinity for hydroxyapatite, a structural component of the bone matrix, and are therefore absorbed by bone tissues. BPs are then able to suppress bone resorption by OCs by inhibiting intracellular mevalonic acid metabolism and inducing apoptosis (10). However, BPs are strong inhibitors of osteoclast function, which can lead to the inhibition of normal bone turnover. In certain cases, this inhibition results in a severe complication associated with the administration of BPs, known as bisphosphonate-associated osteonecrosis of the jaw (11). Salmon calcitonin (sCT) is a classic anti-osteoporosis drug, and is an active peptide, comprised of 32 amino acids with a molecular weight of 3,500 Da (12). Experimental administration of sCT in rats increases cancellous (spongy) bone volume and trabecular number and may reduce the number of osteoclasts (13,14). sCT has been used for the long-term treatment of metabolic bone diseases associated with bone turnover, including Paget’s disease of bone and hypercalcemia associated with bone cancer. sCT rarely causes hypocalcemia and has been used as a routine drug for the clinical treatment of postmenopausal osteoporosis (15).

A daily low dose of aspirin may prevent cardiovascular disease and has few adverse reactions. Solheim *et al* (16) previously revealed that aspirin (ASA), also known as acetylsalicylic acid, may have a role in the inhibition of bone resorption, and subsequent epidemiological reports also found that regular use of low-dose ASA or other NSAIDs is frequently associated with increased bone density (17,18). Through acting on the Fas/FasL signaling pathway, ASA intervenes in the development of osteoporosis by inhibiting the apoptotic effect of T lymphocytes on bone marrow-derived mesenchymal cells, promoting osteogenic differentiation and inhibiting osteoclast differentiation (19,20). Additionally, cyclooxygenase-2 (COX-2) may promote bone resorption by altering the expression of matrix metalloproteinase-1 (MMP-1) and interleukin (IL)-6 (21). ASA acts as an effective inhibitor of COX-2, and may also reduce COX-2, inhibiting the development of bone resorption in osteoporosis (17). Therefore, ASA has significant potential as an effective, cheaper alternative to the drugs currently used to treat osteoporosis.

In the present study, experiments were conducted to assess the effectiveness of combined therapy with sCT and ASA (sCT+ASA) in rats that were ovariectomized (OVX) via bilateral oophorectomy, an animal model that has been accepted as an effective simulation of osteoporosis in postmenopausal women with characteristic bone resorption (22). ASA or sCT was administered to ovariectomized rats as a monotherapy (OVX+sCT or OVX+ASA) or in combination (OVX+sCT+ASA) to inhibit bone resorption. Dual energy X-ray absorptiometry, bone biomechanics and serum markers of bone turnover were evaluated to assess the therapeutic effects of the drug combination, and to study the impact of the drug combination on osteoclast activity and the OPG/RANKL/RANK system *in vivo*.

## Materials and methods

### Experimental design

A total of 40 healthy, female Sprague-Dawley (SD) rats (3-months old, weighing 245–300 g each) were purchased from Guangdong Province Medical Experimental Animal Center (license no. SCXK 2008-0002; Guangdong, China). The rats were reared in an environment with an ambient temperature of 24–26°C, relative humidity of 45–65% and 12 h of daylight with *ad libitum* access to food and water. The care of the experimental animals met the requirements of Guidance Suggestions for the Care and Use of Laboratory Animals, issued by The Ministry of Science and Technology of the People’s Republic of China (http://www.calas.org.cn/html/jypx/zcfg/20111129/1309.html). All surgical and treatment procedures were approved by the Animal Ethics Committee of Guangdong Medical College (Zhanjiang, China).

Following adaptive feeding, according to weight, the rats were divided randomly into five groups (n=8 rats/group): The sham group (Sham), the aspirin group (OVX+ASA or ASA), the calcitonin group (OVX+sCT or sCT), the combined treatment group (OVX+sCT+ASA or sCT+ASA) and the placebo group (OVX). Following an intraperitoneal injection of 7% chloral hydrate (0.5 ml/100 g; Guangzhou Chemical Reagent Factory, Guangzhou, China), rats in the sCT, ASA, sCT+ASA and OVX placebo groups underwent bilateral oophorectomies, while those in the Sham group underwent abdominal incision and suture without concomitant oophorectomy (23). Postoperatively, all rats were provided with free access to food and water. No treatment was administered during the first two weeks following the operation, to allow for the development of osteoporosis. Beginning two weeks post-operation, the groups were administered treatment intervention as follows: i) Rats in the aspirin group (OVX+ASA) were given a gavage of low-dose aspirin (Hunan Xinhui Pharmaceutical Industry Ltd. Co., Hunan, China). The daily dosage of aspirin for rats (per square meter of body surface) was calculated to be similar to that of a low-dose aspirin regimen in humans (34.4 mg/kg/day); ii) rats in the calcitonin group (OVX+sCT) were injected subcutaneously in the neck with 2 U/kg/day sCT (Miacalcic; Beijing Novartis Pharmaceutical Co. Ltd., Beijing, China); iii) rats in the OVX+sCT+ASA group were injected subcutaneously with sCT (2 U/kg/d) and administered a gavage of aspirin (34.4 mg/kg/d) and iv) rats in the Sham and OVX groups were administered a subcutaneous injection of saline (0.5 ml/kg/day) and a gavage of saline (5 ml/kg/day; Guangzhou Jinhuada Chemical Reagent Co., Ltd.).

### Sample collection

All rats were sacrificed 12 weeks following the commencement of treatment intervention. Blood was collected from the right ventricle under anesthesia with intraperitoneal injection of chloral hydrate (0.5 ml/10 g, 7%). The blood samples were placed into a 4°C refrigerator for 3 h and then centrifuged (989 x g, 4°C, 20 min). The resulting serum supernatants were recovered at 10,000 x g, placed in Eppendorf tubes (Thermo Fisher Scientific, Waltham, MA, USA) and stored at −80°C until required for further analysis. Following sacrificing the rats, the right femurs were rapidly removed and the muscles, ligaments and other tissues on the surface were cleaned off. The femurs were then washed with alcohol and rinsed three times with sterile saline. Each end of the femur was cut with sterile surgical instruments under sterile conditions to expose the marrow cavity and 1 ml low-sugar Dulbecco’s modified Eagle’s medium (DMEM; Thermo Fisher Scientific) was drawn with a 2.5-ml disposable sterile syringe (Guangzhou Jinhuada Chemical Reagent Co., Ltd.) to flush the marrow cavity repeatedly. The bone-marrow cell suspension was placed into a 2-ml freezing tube and stored at −80°C until prior to analysis by reverse transcription-quantitative polymerase chain reaction (RT-qPCR). The left femurs and the fourth lumbar vertebrae were removed and, following separation of the surrounding soft tissues, these bones were wrapped with saline-soaked gauze and aluminum foil and stored at −20°C until required for measurements of BMD and bone biomechanics. The third lumbar vertebrae and right tibias were cut from the adhering connective tissues, fixed with (5 ml, 2.5%) paraformaldehyde for 24 h and then soaked in 5 ml of 10% EDTA (Guangzhou Jinhuada Chemical Reagent Co., Ltd.) for decalcification at room temperature. The EDTA solution was replaced every three days, and following three weeks in EDTA, when there was no resistance when using a pin to puncture the bones, the metaphyseal bones were paraffin-embedded and sectioned for hematoxylin and eosin (H&E) staining. The right tibias were sectioned and stored at 4°C for immunohistochemical staining (IHC).

### Serological marker detection

Serum calcium (Ca), phosphorus (P) and alkaline phosphatase (ALP) concentrations were determined using an automated biochemical analyzer (Olympus AU2700; Olympus, Tokyo, Japan) at the Affiliated Hospital of Guangdong Medical College (Zhanjiang, China). Serum concentrations of proteins were determined using ELISA kits (BGP ELISA kit; PICP ELISA kit and ICTP ELISA kit; RapidBio, West Hills, CA, USA): Osteocalcin/bone γ-carboxyglutamic-acid (GLA)-containing proteins (OC), procollagen I C-terminal peptide (PICP) and type I collagen cross-linked telopeptide (ICTP) expression was evaluated.

### Measurement of BMD and bone mechanics

The left femurs and fourth lumbar vertebrae were sent to the Department of Nuclear Medicine, Guangzhou Overseas Chinese Hospital (Guangzhou, China), affiliated to Southern Medical University (Guangzhou, China), for examination by Lunar Prodigy dual-energy X-ray absorptiometry (DXA; GE Healthcare Life Sciences, Little Chalfont, UK). The placement positions of each femur and lumbar were consistent. The scanning results were analyzed using the small animal software supplied (combined with the Lunar Prodigy dual energy X-ray; GE Healthcare, Madison, WI, USA). Subsequently, the specimens were soaked in 50% alcohol, the femurs were assessed using a three-point stress test and the bone biomechanics of the fourth lumbar vertebrae were assessed with a lumbar compression test.

### Isolation of RNA and RT-qPCR analysis

Total RNA was extracted from the cryopreserved bone marrow cells, which were ground in liquid nitrogen into fine powders using TRIzol^®^ reagent (Invitrogen Life Technologies, Carlsbad, CA, USA). β-actin was used as an internal control. PCR primer sets (Thermo Fisher Scientific) were designed and synthesized for the three proteins as follows: OPG forward, 5′-GCCCAGACGAGATTGAGAGA-3′ and reverse, 5′-ACGGTTTTGGGA AAGTGGTA-3′; RANKL forward, 5′-CCGTGCAAAGGG AATTACAA-3′ and reverse, 5′-GGATGTCGGCAGCAT TGAT-3′; β-actin forward, 5′-AGGGAAATCGTGCGT GACAT-3′ and reverse, 5′-AGGGAAATCGTGCGTGA CAT-3′. The RT conditions were as follows: 42°C for 1 h and 0°C for 2 min (HiScript^®^ 1st Strand cDNA Synthesis kit; Vazyme Biotech Co., Ltd., Nanjing, China). Using these primer sets, the predicted amplicons for OPG, RANKL and β-actin were 160, 150 and 150 bp, respectively. SYBR^®^ Green-labeled products were used (Bio-Rad Laboratories (Shanghai) Co., Ltd., Guangzhou, China). The incorporation of SYBR Green into the PCR products was monitored in real-time following each PCR cycle, resulting in the calculation of the threshold cycle (Ct value), which defines the PCR cycle number at which the exponential growth of PCR products begins.

The PCR cycling conditions were as follows: Initial denaturation at 95°C for 5 min, followed by 40 cycles of denaturation at 95°C for 15 sec, annealing at 60°C for 15 sec and extension at 72°C for 32 sec (72°C for 32 sec also facilitated detection of the fluorescence signal following each round of extension). A melting curve analysis from 60 to 95°C was also performed. Gene expression of OPG and RANKL was quantified relative to β-actin as the internal control.

### H&E staining and immunohistochemistry

The third lumbar vertebrae and right tibias underwent gradient ethanol dehydration, and were then embedded in paraffin and cut into 5-µm sections for H&E staining (Guangzhou Jinhuada Chemical Reagent Co., Ltd.). Following H&E staining, the sections were observed using a Nikon E400 low-magnification microscope (x40; Nikon Corp., Tokyo, Japan), and images were captured with the DS-U1 digital camera built into the microscope.

Right tibia slices were immunohistochemically stained according to a previously described protocol (24), and images were recorded with a x250 magnification microscope camera (Olympus DP71; Olympus). The staining intensity was assessed using Image-Pro Plus version 6.0 (Media Cybernetics, Rockvilled, MD, USA). The antibodies used were as follows: OPG primary antibody (1:80; BA1475; Wuhan Boster Biological Technology Co. Ltd., Wuhan, China; rabbit anti-rat OPG), RANKL primary antibody (1:80; BA1323; Wuhan Boster Biological Technology Co. Ltd.; rabbit anti-rat OPG ligand) and rabbit anti-rat biotinylated secondary antibody (SP-0023; Beijing Biosynthesis Biotechnology Co., Ltd., Beijing, China). The antibodies used were as follows: OPG primary antibody (rabbit anti-rat OPG polyclonal antibody at a dilution of 1:80, incubated overnight at 4°C; cat. no. BA1475; Wuhan Boster Biological Technology Co., Ltd., Wuhan, China); RANKL primary antibody (rabbit anti-rat OPG ligand polyclonal antibody at a dilution of 1:80, incubated overnight at 4°C; cat. no. BA1323; Wuhan Boster Biological Technology Co. Ltd.) and rabbit anti-rat biotinylated secondary antibody (at a dilution of 1:1,000 and incubated for 2 h at 37°C; cat. no. SP-0023; Beijing Biosynthesis Biotechnology Co., Ltd., Beijing, China).

### Statistical analysis

All experimental results are expressed as the mean ± standard deviation, and were processed using SPSS software version 17.0 for Windows (SPSS, Inc., Chicago, IL, USA). All data were initially tested for normality and homogeneity of variance. Data among the groups were compared using one-way analysis of variance and Student-Newman-Keuls analysis. P<0.05 was considered to indicate a statistically significant difference.

## Results

### Combined treatment significantly enhances BMD in OVX rats

**Figure 1:**
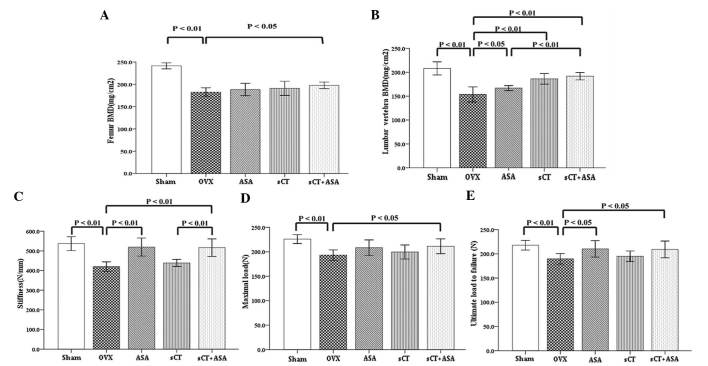
Combined treatment (sCT+ASA) enhances BMD and femur bone biomechanics. Comparison of the effects of ASA, sCT and combined treatment (sCT+ASA) on BMD and mechanical strength in OVX rats. (A) BMD of right femur; (B) BMD of fourth lumbar vertebra; (C) femur stiffness; (D) femur maximal load and (E) femur ultimate failure load. Values are expressed as the mean ± standard deviation. P-values were calculated according analysis of variance with Student-Newman-Keuls analysis. ASA, aspirin; sCT, salmon calcitonin; BMD, bone mineral density; OVX, ovariectomized.

At 14 weeks post-surgery, BMD in the femur (Fig. 1A) and fourth lumbar spine (Fig. 1B) of the OVX group was decreased, by 24.48% and 26.18%, respectively, compared with that of the Sham group (P<0.01). Compared with the OVX group at 12 weeks following the commencement of treatment, rats that received ASA or sCT monotherapy demonstrated no significant differences in the BMD of the femur, while the combined sCT+ASA therapy resulted in an 8.42% increase in BMD (P<0.05). Compared with the OVX group, the BMD of the fourth lumbar spine was increased by administration of ASA alone (P<0.05) and significantly increased by a single injection of sCT; however, there was no significant difference detected between the OVX+ASA and OVX+sCT groups. With the combination treatment of sCT+ASA, the BMD of the fourth lumbar spine was significantly increased, by up to 24.90%, compared with that of the OVX group, and greater than that of the OVX+ASA group (P<0.05).

### Combined treatment enhances femur bone biomechanics in OVX rats

At 14 weeks post-surgery, all measurements of femur bone biomechanics, including stiffness (Fig. 1C), maximal load (Fig. 1D) and ultimate failure load (Fig. 1E), were decreased in the OVX group compared with those of the Sham group (all P<0.01), demonstrating the validity of the OVX rat model for osteoporosis. Compared with the OVX group, at 12 weeks following commencement of therapy, rats in the OVX+sCT group indicated no significant improvement in femur bone biomechanics (P>0.05), while rats in the OVX+ASA group benefited from an increase the femur ultimate failure load (P<0.05) and a significant increase in stiffness (P<0.01). Compared with the OVX group, combination therapy in the OVX+sCT+ASA group increased the ultimate failure load and maximal load of the femur (P<0.05) and significantly improved femur stiffness in comparison to that of the sCT group (P<0.05). As with measurements of femur bone biomechanics, all OVX rats demonstrated significant losses in bone strength of the fourth lumbar vertebrae, measured by stiffness, maximal load and ultimate load compared with those of the Sham group; however, none of the treatment groups exhibited any significant improvements in vertebral bone strength compared with that of untreated OVX rats (data not shown).

### Combined treatment rescues bone morphology of OVX rats

**Figure 2:**
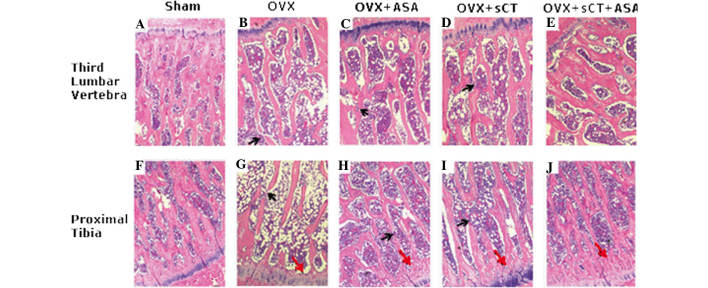
Combined treatment (ASA+sCT) ameliorates the morphological changes induced in OVX rats. Histological analysis of effects of ASA, sCT and combined treatment (sCT+ASA) on the third lumbar vertebrae and proximal tibia of OVX rats. Specimens were stained with hematoxylin and eosin (magnification, x40). Sections of third lumbar vertebrae tissues of the (A) Sham group; (B) OVX group; (C) OVX+ASA group; (D) OVX+sCT group and (E) OVX+sCT+ASA group. Sections of proximal tibia of the (F) Sham group; (G) OVX group; (H) OVX+ASA group; (I) OVX+sCT group and (J) OVX+sCT+ASA group. ASA, aspirin; sCT, salmon calcitonin; BMD, bone mineral density; OVX, ovariectomized.

The third lumbar vertebrae and the right proximal tibia of rats in all groups underwent H&E staining to facilitate the observation of histological changes (Fig. 2). The cancellous bones of the rats in the Sham group demonstrated neatly arranged and normal bone trabeculae, which were bulky, plump and connected to a network (Fig. 2A and F). The bone trabeculae of rats in the OVX group were arranged sparsely, were disorderly and indicated significant resorption, were thinner or had disappeared altogether and the remaining connections were incomplete (black arrow; Fig. 2B and G). Distortions and fractures were common, the gaps became larger and the bone layer under the osteoepiphysis was markedly thinner (red arrow; Fig. 2B and G). The bone trabecula arrangement in rats in the OVX+ASA group was improved compared with that of the OVX group; the number of trabecula was increased and trabecular connections were enhanced. However, the trabecular rods in the OVX+ASA group were slightly smaller, trabecular continuity was not significantly improved and there were still apparent signs of resorption, fractures and incomplete connections (black arrow). The bone layer under the osteoepiphysis was recovered to a certain extent, but had not reached normal levels (red arrow; Fig. 2C and H).

The bone trabeculae of rats in the OVX+sCT group were more orderly arranged than those in the OVX group. The trabecular number in the OVX+sCT group was not markedly increased, but the trabecular bones were significantly thicker, although still prone to fracture and resorption (black arrow). The bone layer under the osteoepiphysis in rats treated with sCT was recovered to a certain extent (red arrow; Fig. 2D and I).

Compared with monotherapy and combined groups, the trabecular bones were thickened, the number was increased and the connections were compact. Fractures and absorption were reduced and the bone layer under the osteoepiphysis was also improved to a certain extent (red arrow; Fig. 2E and J). The bone trabeculae of rats in the OVX+sCT+ASA group were more neatly and uniformly arranged, compared with those in the OVX group, the structure was clear and the network structure and trabecular morphology was almost fully recovered. Furthermore, the trabecular bones were thickened, the number was increased, and the connections were compact. Fractures and absorption were reduced, and the bone layer under the osteoepiphysis was also improved to a certain extent (red arrow; Fig. 2E and J).

### Combined treatment rescues expression of bone metabolic biomarkers of OVX rats

**Figure 3:**
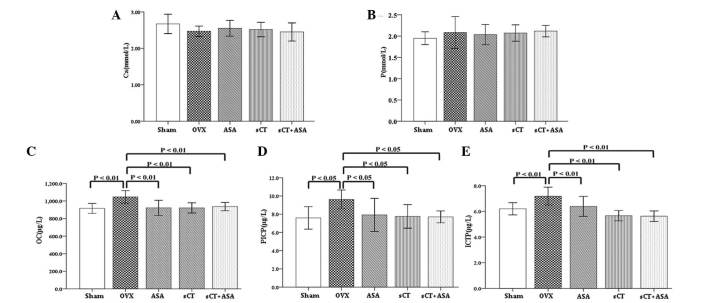
Combined treatment (ASA+sCT) reverses alterations in serum parameters induced in OVX rats. Effects of ASA, sCT and combined treatment (sCT+ASA) on serum parameters of OVX rats. Serum Ca and P concentrations were determined using an automated biochemical analyzer (Olympus AU2700), and serum concentrations of proteins were determined by ELISA. Values are expressed as the mean ± standard deviation. P-values were calculated by analysis of variance with Student-Newman-Keuls analysis. (A) Ca; (B) P; (C) OC; (D) PICP and (E) ICTP. ASA, aspirin; sCT, salmon calcitonin; BMD, bone mineral density; OVX, ovariectomized; OC, osteocalcin; PICP, procollagen type I C-terminal propeptide; ICTP, type I collagen cross-linked telopeptide.

The serological analysis results of the various groups are shown in Fig. 3. There was no significant difference in serum Ca and P levels amongst any group (P>0.05; Fig. 3A and B). As shown in Fig. 3C–E and Table I, levels of serum bone metabolism indicators, including ALP, OC, PICP and ICTP, were significantly upregulated in the OVX group compared with those of the Sham group (P<0.05 for OC; P<0.01 for PICP and ICTP). A significant decline in ALP levels was detected in all the treated groups, compared with those of the OVX group (P<0.05); whereas compared with the Sham group, ALP was elevated in all treatment groups (P<0.05) and increased most markedly in the OVX+sCT+ASA group. Compared with the OVX group, serum levels of OC, PICP and ICTP in each treatment group were decreased (P<0.05 for OC; P<0.01 for PICP and ICTP), and the largest decrease was observed in the combined treatment group. There were no statistically significant differences detected in the serum levels of OC, PICP or ICTP between any treatment group and the Sham group.

### Combined treatment attenuates the decrease in OPG/RANKL ratio induced in OVX rats

**Figure 4:**
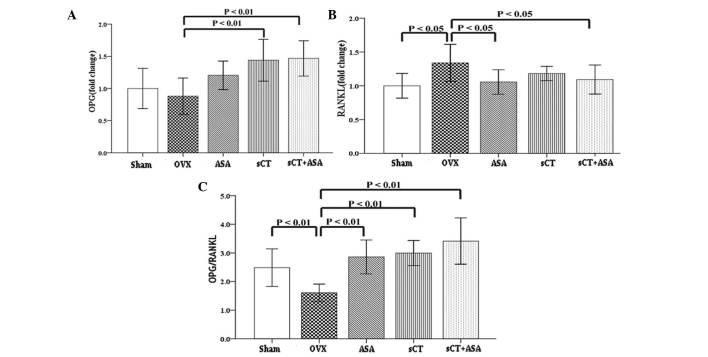
Combined treatment (sCT+ASA) enhances the OPG/RANKL expression ratio in OVX rats. Effects of ASA, sCT and combined treatment (sCT+ASA) on mRNA levels in femur bone marrow cells from ovariectomized (OVX) rats. Gene expression was analyzed by reverse transcription-quantitative polymerase chain reaction. Values are expressed as the mean ± standard deviation. P-values were calculated by analysis of variance with Student-Newman-Keuls analysis. (A) OPG; (B) RANKL; (C) mRNA expression ratio of OPG/RANKL. ASA, aspirin; sCT, salmon calcitonin; OVX, ovariectomized; OPG, osteoprotegerin; RANKL, receptor activator of nuclear factor κB ligand; mRNA, messenger RNA.

In order to evaluate the changes in gene expression of OPG and RANKL following ovariectomy and drug treatments of OVX rats, RT-qPCR was used to measure the relative quantities of their mRNA expression levels in bone marrow cells obtained from rat femurs (Fig. 4). As shown in Fig. 4B, the mRNA expression of RANKL was increased significantly in rats of the OVX group (P<0.05 vs. the Sham group). However, no significant differences were observed in OPG gene expression between the Sham and OVX groups (P>0.05; Fig. 4A). The OPG/RANKL ratio, an index of osteoclastogenic inhibition, was significantly decreased following ovariectomy (P<0.01 vs. Sham group; Fig. 4C). The mRNA expression of RANKL was significantly decreased in the OVX+ASA and OVX+sCT+ASA groups, compared with that of the Sham group (P<0.05; Fig. 4B). However, no significant difference was observed in OVX+sCT rats (P>0.05; Fig. 4B). The mRNA expression of OPG was increased significantly following sCT and sCT+ASA treatments (P<0.01; Fig. 4A), and compared with that of the OVX group, mRNA expression of OPG was increased with ASA treatment, but the difference was not significant (P>0.05; Fig. 4A). The OPG/RANKL ratio increased with all drug treatments (P<0.01; Fig. 4C), and the ratios were similar between the ASA and sCT groups. The combination treatment (sCT+ASA) induced the highest OPG/RANKL ratio, but there was no statistically significant difference between this ratio and that of the other treatment groups.

### Combined treatment rescues RANKL and OPG protein expression in the proximal tibiae

**Figure 5:**
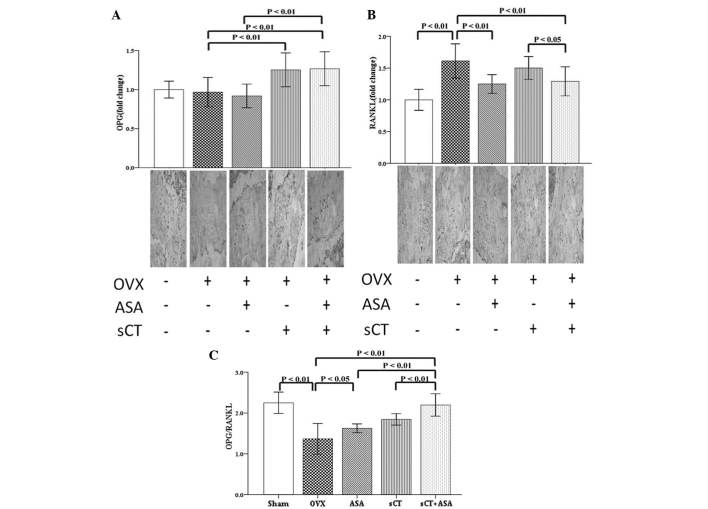
Combined treatment significantly enhances the OPG/RANKL ratio in the proximal tibia of OVX rats. Effects of ASA, sCT and combined treatment (sCT+ASA) on protein expression levels in the proximal tibia of OVX rats. Specimens were observed at magnification, x250. Antigens were detected using an avidin-biotin-peroxidase system and visualized with 3,3′-diaminobenzidine. Protein levels were measured by densitometry. Values are expressed as the mean ± standard deviation. P-values were calculated by analysis of variance, with Student-Newman-Keuls analysis). (A) OPG; (B) RANKL; (C) OPG/RANKL protein ratio. Positive staining for OPG and RANKL was mainly localized in the chondrocytes of the epiphyseal growth plate and trabeculae of bone. ASA, aspirin; sCT, salmon calcitonin; OVX, ovariectomized; OPG, osteoprotegerin; RANKL, receptor activator of nuclear factor κB ligand.

Cells positive for OPG and RANKL exhibited brown staining by IHC in the proximal tibiae of all groups. Significant RANKL and OPG protein staining was observed in all chondrocytes of the epiphyseal growth plate, including the articular cartilage. Control sections that were stained with the secondary antibody only exhibited no staining, confirming the specificity of the response (Fig. 5). IHC staining revealed that the levels of RANKL in the right proximal tibia of the OVX group were increased significantly, compared with those of the Sham group (P<0.01; Fig. 5B). However, no difference in OPG protein expression was detected between the two groups (P>0.05; Fig. 5A). These results indicated that the OPG/RANKL ratio was significantly decreased following ovariectomy (P<0.01; Fig. 5C). None of the parameters tested differed significantly between the sCT-treated group and the OVX group (P>0.05; Fig. 5B). In the OVX+ASA and OVX+sCT+ASA groups, the levels of RANKL in proximal tibia were decreased significantly, when compared with those of the OVX group (P<0.01; Fig. 5B). In the OVX+sCT and OVX+sCT+ASA groups, the levels of OPG in the proximal tibia were significantly increased, compared with those of the OVX group (P<0.01; Fig. 5A). No significant differences in OPG protein expression were observed in the ASA group (P>0.05; Fig. 5A). The lower OPG/RANKL ratio detected in the OVX group was reversed in all of the treatment groups, but the OPG/RANKL ratio was highest in the OVX+sCT+ASA (Fig. 5C).

## Discussion

Following the increase in the numbers and proportion of the elderly population in more economically developed countries, osteoporosis has become a significant global public health concern. Once osteoporotic fractures occur, they can result in substantial economic losses and emotional burden to the affected individuals and their families. Furthermore, since much of the cost of healthcare for elderly patients is borne by government agencies, there is a significant societal burden associated with osteoporosis. Nasal sprays containing sCT are relatively inexpensive and have previously been approved for use in the treatment of osteoporosis in multiple countries; however, recently two experts at the US Food and Drug Association have recommended that the marketing of sCT for osteoporosis be stopped due to a lack of demonstrated efficacy (25). The most effective bone-sparing drugs for use in the treatment of osteoporosis tend to be relatively expensive and may only be approved by insurers in patients with more severe symptoms of OA (26). Therefore, the development of cost-effective treatments for osteoporosis is a high priority for biomedical research.

It has previously been reported that once female rats are ovariectomized, the major manifestations of osteoporosis observed are an increase in the number of osteoclasts, enhanced osteolysis and high-turnover bone metabolism, with bone resorption surpassing bone formation. Thus, there are numerous similarities between the alterations to bone metabolism observed in human postmenopausal osteoporosis and those of ovariectomized (OVX) rats, and OVX rats are therefore considered an effective animal model of osteoporosis in menopausal women (22,27). The current study was designed to explore the use of combined therapy of sCT and ASA to prevent bone loss in estrogen-deficient rats. OVX rats were treated with a combination of sCT and ASA for 12 weeks. The results revealed that the strength of the femoral shaft bone was increased (but not the load-bearing capacity of the lumbar spine) and trabecular bone structure had been improved. The combination treatment (sCT+ASA) was also able to reduce the expression of bone turnover markers OC, PICP and ICTP. It was also found that sCT+ASA increased OPG gene expression and reduced RANKL gene expression in bone marrow cells.

BMD measurement is considered to be the standard test for the diagnosis of osteoporosis and DXA has been used in previous studies to measure BMD in rats (28,29). In the present study, at 14 weeks following ovariectomy, the lumbar spine and femoral BMD in OVX rats was significantly decreased, compared with that of the Sham group (P<0.01), indicating that the bone mass was reduced. Combined treatment of OVX rats with sCT+ASA increased the bone mass in the lumbar spine (P<0.01) and femur (P<0.05), compared with that of the OVX group. However, when sCT or ASA treatment was used alone, femoral BMD was not significantly improved (P>0.05). This may have been due to the major part of the femur being composed of compact bone, and the change or loss of compact bone in the femur following ovariectomy was slow. Therefore, when dual-energy X-ray was used to measure the total femur BMD, following treatment with sCT or ASA alone, the change in femoral BMD was smaller. Similarly to the results of a study by Kavuncu *et al* (30), sCT treatment alone increased rat lumbar spine BMD (P<0.01), while ASA treatment alone did not (P<0.05).

BMD can only indicate the degree of bone mineralization, which represents one component of bone strength (31). Bone strength is also reflected by bone quality. Bone quality parameters include damage accumulation, bone microarchitecture and bone mineralization (32). The combined sCT+ASA treatment enhanced femur stiffness (P<0.01), maximal load (P<0.05) and ultimate failure loads (P<0.05). ASA treatment increased femoral stiffness (P<0.01) and ultimate failure load (P<0.05), perhaps as ASA was able to improve the bone trabeculae and increase the quantity of cortical bone in OVX rats (20), while sCT treatment alone failed to enhance the various parameters of the femur. In humans, calcitonin (CT) treatment alone is not effective in the long-term reduction of hip fracture risk; this may be due to the fact that the metabolism of cancellous bone in humans is more vulnerable to the impact of bone metabolism changes than cortical bone (33,34). However, in OVX rats, sCT appears to be effective in the prevention of cancellous bone loss (35).

Whether sCT and ASA were used alone or in combination, there was no significant effect on the biomechanical parameters of the rat lumbar bone. There are several reasons that may explain this result. Rat lumbar motion differs from that of human upright walking, therefore the rat lumbar spine is not the main axial load-bearing bone. In addition, the rat lumbar compression test indicates that there may be certain defects in the drug treatments. The ability of bone to withstand pressure also depends on the quality and arrangement of the trabecular bone (36,37). Histological methods indicated that the trabeculae of cancellous bones in OVX rats were sparse, fractured and disordered in arrangement. At 12 weeks post-treatment with sCT and ASA, the trabecular bone structure in the tibia and lumbar spine was significantly improved compared with that of treatment with either drug alone.

DXA measurement is considered to be the ‘gold standard’ for the diagnosis of osteoporosis, recognized by the international academic and medical communities. DXA may directly reflect the BMD, but not all individuals with low BMD have fractures, and relying solely on BMD changes underestimates the degree of osteoporotic fracture risk (38). Bone turnover markers, which do not depend upon BMD, have been widely used to assess bone reconstruction and to assess the effects of drugs on bone metabolism *in vivo*. The biochemical indicators primarily used for evaluating bone formation are OC and PICP, and a marker of bone resorption is ICTP. The results of the present study indicated that the levels of OC (P<0.01), PICP (P<0.05) and ICTP (P<0.01) were all increased in the OVX group. This may be due to the lack of estrogen resulting in hyperactivity in osteoclastic bone resorption and leading to a subsequent increase in bone resorption markers (39). Regardless of whether sCT and ASA were used alone or in combination, they were able to inhibit the high bone turnover observed in OVX rats; however, there was no significant difference between the effectiveness of the treatments, alone or in combination.

During the processes of macrophage/monocyte differentiation into osteoclasts and osteoclast activation, the differentiation and maturation of osteoclasts are inseparable from activation of the OPG/RANKL/RANK system (40). When osteoporosis occurs, there are differing levels of disorder detected in the expression of OPG and RANKL (41). Previous studies have found that a calcitonin receptor-like receptor (CLR) is expressed on the osteoblast surface (42) and that CLR has a high affinity for calcitonin gene-related peptide (CGRP), which can exhibit pharmacological effects similar to those of CT (43). CT promotes cartilage formation through the ERK1/2 signaling pathway (44), and CGRP is able to promote the expression of the osteoblast OPG gene (45). In aged rats administered an agonist of prostaglandin E, RANKL gene expression in the rat vertebrae was increased, while OPG was unaffected. In cultured bone marrow cells *in vitro*, prostaglandin E agonists increased RANKL gene expression in bone marrow cells and reduced OPG gene expression (46). Prostaglandin production is primarily mediated by COX-2 in osteoblasts (47). ASA as a non-selective COX inhibitor and is therefore able to inhibit prostaglandin production. Long-term ASA treatment may systemically alter multiple serum markers, reducing RANKL and increasing OPG in OVX mice.

In the current study, it was demonstrated that once OVX rats were treated with a combination of sCT and ASA, OPG gene expression in bone marrow cells and OPG protein expression in the tibia metaphysis were increased (P<0.01), while RANKL gene expression in bone marrow cells (P<0.05) and RANKL protein expression in the tibial metaphysis (P<0.01) were reduced. In addition, the OPG/RANKL ratio, which reflects osteoblast activity, was increased (P<0.01). However, following treatment with ASA alone, it was found that only RANKL expression was decreased (P<0.05), while OPG expression was unaffected (P>0.05). Following sCT treatment alone, it was found that only OPG expression was increased (P<0.01) and RANKL expression was unaffected (P>0.05).

The results of the present study demonstrated that sCT (2 U/kg/d) and ASA (34.4 mg/kg/d) in combination prevented trabecular bone loss in OVX rats (as evidenced by a higher BMD in the femur and vertebrae), improved the structure of the trabecular bone in vertebrae, increased femoral shaft strength and reduced serum markers of bone turnover. The combined therapy increased OPG gene expression in bone marrow cells and OPG protein expression in the tibial metaphysis in OVX rats, while reducing RANKL gene expression in bone marrow cells and RANKL protein expression in the tibial metaphysis, resulting in a significant elevation of the OPG/RANKL ratio (P<0.01). At the protein level, it was apparent that sCT and ASA *in vivo* impacted distinct factors in the OPG/RANKL/RANK pathway to inhibit bone resorption: ASA monotherapy only lowered RANKL protein expression in bone marrow cells, while CT monotherapy only increased OPG protein expression. However, combination therapy (sCT+ASA) lowered RANKL protein expression and increased OPG protein expression. These results suggested a synergistic effect of the two drugs that may explain the superior performance of combination therapy in increasing femur bone strength, normalizing morphology of the trabecular networks in vertebrae and increasing BMD in the femurs and vertebrae.

It was therefore hypothesized that sCT and ASA combination therapy may regulate osteoclast activity and inhibit bone resorption through the OPG/RANKL/RANK pathway, and these experiments may provide the basis for future clinical trials. ASA is cheap, sCT is relatively inexpensive and ASA+sCT combined therapy is suggested to have fewer adverse reactions compared with bisphosphonates or hormone-replacement therapies. However, the safety and efficacy of ASA+sCT combination therapy requires further assessment in clinical trials.

## Figures and Tables

**Table I:** Effects of combined ASA and sCT treatment on serum parameters.

Serum component	Sham	OVX	OVX+ASA	OVX+sCT	OVX+sCT+ASA
Ca (mmol/l)	2.67±0.27	2.47±0.14	2.55±0.22	2.52±0.20	2.45±0.25
Pi (mmol/l)	1.95±0.15	2.09±0.37	2.04±0.24	2.07±0.19	2.12±0.13
ALP (U/l)	87.65±32.99	202.96±55.16^b^	126.67±32.17^ad^	136.93±18.60^ad^	148.34±29.07^bd^
PICP (µg/l)	7.59±1.24	9.62±1.04^a^	7.92±1.81^c^	7.76±1.29^c^	7.71±0.65^c^
ICTP (µg/l)	6.20±0.48	7.19±0.70^b^	6.39±0.78^d^	5.67±0.40^d^	5.62±0.41^d^

Values are expressed as the mean ± standard deviation (n=8).

a P<0.05 vs. Sham;

b P<0.01 vs. Sham;

c P<0.05 vs. OVX;

d P<0.01 vs. OVX; P-values were calculated using analysis of variance with Student-Newman-Keuls analysis. ASA, aspirin; sCT, salmon calcitonin; OVX, ovariectomized Ca, calcium; Pi, inorganic phosphorus; ALP, alkaline phosphatase; PICP, procollagen type I C-terminal propeptide; ICTP, type I collagen cross-linked telopeptide.
